# Structural Properties and Energy Spectrum of Novel GaSb/AlP Self-Assembled Quantum Dots

**DOI:** 10.3390/nano13050910

**Published:** 2023-02-28

**Authors:** Demid S. Abramkin, Mikhail O. Petrushkov, Dmitrii B. Bogomolov, Eugeny A. Emelyanov, Mikhail Yu. Yesin, Andrey V. Vasev, Alexey A. Bloshkin, Eugeny S. Koptev, Mikhail A. Putyato, Victor V. Atuchin, Valery V. Preobrazhenskii

**Affiliations:** 1 Laboratory of Molecular-Beam Epitaxy of A3B5 Compounds, Institute of Semiconductor Physics, SB RAS, Novosibirsk 630090, Russia; 2 Department of Physics, Novosibirsk State University, Novosibirsk 630090, Russia; 3 Laboratory of Physical Bases of Semiconductor Heterostructures Epitaxy, Institute of Semiconductor Physics, SB RAS, Novosibirsk 630090, Russia; 4 Laboratory of Molecular-Beam Epitaxy of Elementary Semiconductors and A3B5 Compounds, Institute of Semiconductor Physics, SB RAS, Novosibirsk 630090, Russia; 5 Laboratory of Nonequilibrium Semiconductor Systems, Institute of Semiconductor Physics, SB RAS, Novosibirsk 630090, Russia; 6 Department of Automation and Computer Engineering, Novosibirsk State Technical University, Novosibirsk 630073, Russia; 7 Laboratory of Optical Materials and Structures, Institute of Semiconductor Physics, SB RAS, Novosibirsk 630090, Russia; 8 Research and Development Department, Kemerovo State University, Kemerovo 650000, Russia; 9 R&D Center “Advanced Electronic Technologies”, Tomsk State University, Tomsk 634034, Russia; 10 Department of Industrial Machinery Design, Novosibirsk State Technical University, Novosibirsk 630073, Russia

**Keywords:** quantum dots, GaSb/AlP, molecular beam epitaxy, structural properties, energy spectrum, QD-Flash

## Abstract

In this work, the formation, structural properties, and energy spectrum of novel self-assembled GaSb/AlP quantum dots (SAQDs) were studied by experimental methods. The growth conditions for the SAQDs’ formation by molecular beam epitaxy on both matched GaP and artificial GaP/Si substrates were determined. An almost complete plastic relaxation of the elastic strain in SAQDs was reached. The strain relaxation in the SAQDs on the GaP/Si substrates does not lead to a reduction in the SAQDs luminescence efficiency, while the introduction of dislocations into SAQDs on the GaP substrates induced a strong quenching of SAQDs luminescence. Probably, this difference is caused by the introduction of Lomer 90°-dislocations without uncompensated atomic bonds in GaP/Si-based SAQDs, while threading 60°-dislocations are introduced into GaP-based SAQDs. It was shown that GaP/Si-based SAQDs have an energy spectrum of type II with an indirect bandgap and the ground electronic state belonging to the X-valley of the AlP conduction band. The hole localization energy in these SAQDs was estimated equal to 1.65–1.70 eV. This fact allows us to predict the charge storage time in the SAQDs to be as long as >>10 years, and it makes GaSb/AlP SAQDs promising objects for creating universal memory cells.

## 1. Introduction

The systems for long-term information storage with the possibility of fast access [1,2] are important for the development of computing technologies. The so-called universal memory cells combining the fast data access peculiar to the dynamic random-access memory (DRAM) and non-volatile long-term data storage will provide a significant increase in the performance and energy efficiency of memory elements that opens up prospects for a revolution in computer architecture. One of the promising methods in this research field is the fabrication of flash memory devices using arrays of III-V semiconductor self-assembled quantum dots (SAQDs) as a floating gate [2,3,4]. SAQDs are self-organized islands of narrow-gap material grown in a wide-gap material matrix [5,6,7,8,9,10,11]. Epitaxial SAQD formation occurs in Stranski–Krastanov growth mode as a result of surface relief reorganization. One of the crucial advantages of this self-organized growth mode is the formation of a nanoscale object array without using nanoscale lithography. The flexibility of the SAQD energy spectrum, caused by the discontinuity of the energy bands at the SAQD/matrix interface, ensures the charge carrier localization in the SAQD. The SAQD sizes are comparable to the de Broglie charge carrier wavelength in III–V semiconductor crystals, and that leads to a complete three-dimensional charge carrier localization in the SAQD. All this makes it possible to reach efficient non-volatile storage of charge carriers in the SAQD. Note, the quantum confinement effect makes low-dimensional HSs such as SAQDs, quantum wells, quantum wires, and other useful materials in such areas of modern optoelectronics such as the fabrication of effective light emitters [12,13,14,15,16] and solar photovoltaics [17,18].

The charge carriers (holes) can be stored in SAQDs and have their effect on the field-effect transistor (FET) channel conductance, as was shown in [3,4], where the first prototypes were studied for the high-temperature flash memory based on InAs/AlGaAs SAQDs. The advantages of the approach of using heterostructures (HSs) based on III–V materials in comparison with a traditional Si/SiO_x_ flash memory were also clearly evaluated. First, the prototypes are characterized by a fast write/erase time (of the order of 10–20 ns), which is comparable to a DRAM. On the one hand, this is caused by the direct capture of holes in the SAQD in the writing mode, and, on the other hand, by the tunnel ejection of holes from the SAQD in the erase mode [19]. The write rate is limited by the recharge rate of the HS capacitance (*f*_cutoff_ ~ 1/(RC)) and the rate of hole capture in SAQDs, and that is characterized by a time of about 1 ps [20]. Second, the presence of hot charge carriers in traditional Si/SiO_x_ flash memory cells leads to dielectric damage, which is expressed in the appearance of leaks and limits the maximum number of rewrite cycles to about 10^6^. The absence of an energy barrier at the SAQD/matrix interface in the write mode makes it possible to avoid the use of hot charge carriers in the writing mode. This is expected to significantly increase the endurance of memory cells. In addition, it is worth noting the superiority of III–V FETs over silicon ones in terms of the charge carrier mobility in the channel [21]. This allows hoping for an acceleration of the data reading procedure in memory cells based on III–V materials.

Unfortunately, despite all the demonstrated advantages, the weak point of a flash memory based on III–V SAQDs is a currently insufficient charge storage time in SAQDs. Indeed, the charge storage time in the prototypes discussed in [4] is just a few milliseconds. This is caused by low hole localization energy *E*_loc_ in SAQDs (<0.8 eV). Thus, the search for novel SAQD heterosystems characterized by a large *E*_loc_ value is topical. In this way, such well-known SAQDs as GaSb/GaAs [22,23,24], as well as relatively novel SAQDs, such as GaSb/AlGaAs [25,26], GaSb/AlAs [27], InSb/AlAs [28], InGaAs/GaP [29,30], GaSb/GaP [31,32], and InGaSb/GaP [33,34] were studied. The best results were achieved for the GaSbP/GaP SAQDs, where *E*_loc_ reached the value of 1.18 eV [32], which provided a data storage time of about 4 days. Unfortunately, this is still not enough for non-volatile memory cells. A promising way to increase *E*_loc_ is to replace the matrix material from GaP to AlP. Since the AlP valence band top lies almost 500 meV lower than that of GaP [35], a significant increase in *E*_loc_ can be assumed. As it was shown by our calculations [36], SAQDs formed in the GaSb/AlP heterosystem can be characterized by the *E*_loc_ value of up to 2.04 eV.

In this work, we first discuss the results of studies of the formation, structural properties, and energy spectrum of GaSb/AlP HSs with SAQDs. The growth of HSs with SAQDs was carried out by molecular-beam epitaxy (MBE) on matched GaP and artificial GaP/Si substrates. The SAQDs formation and crystal structure were studied by reflection high-energy electron diffraction (RHEED) and atomic force microscopy (AFM). The study of the SAQDs energy spectrum was carried out by steady-state photoluminescence spectroscopy (PL) and supplemented by theoretical calculations. It was found that, regardless of the substrate type, Ga_x_Al_1−x_Sb SAQDs are formed. The elastic strain in the SAQDs is almost completely relaxed, while the fraction of Al atoms in the SAQD composition does not exceed 10%. The strain relaxation in GaP/Si-based SAQDs does not lead to a decrease in the SAQDs’ luminescence efficiency. Comparatively, the introduction of dislocations into GaP-based SAQDs leads to a strong decrease in the SAQDs’ luminescence efficiency. Probably, this is caused by the appearance of Lomer 90°-dislocations without uncompensated atomic bonds in GaP/Si-based SAQDs, while threading 60°-dislocations are introduced into GaP-based SAQDs. The studies of SAQDs energy structure showed that they are characterized by an energy spectrum of type II, with the ground electronic state lying in the *X* valley of AlP and the ground hole state lying in Ga_x_Al_1−x_Sb. The *E*_loc_ value was estimated as 1.65–1.70 eV, so charge storage in SAQDs for a long time (>>10 years) is expected [37].

## 2. SAQDs Heterostructure Growth

The HSs were grown by MBE on matched GaP substrates with a (100) crystallographic orientation, as well as on artificial GaP/Si substrates of the same crystallographic orientation. A small, about 0.1%, mismatch between the AlP and GaP lattice constants [35], makes it possible to grow pseudomorphic AlP layers on GaP. The artificial GaP/Si substrates were used to clarify the question about a possible monolithic integration of the GaSb/AlP heterostructures with SAQDs and Si substrates. The monolithic integration of III–V HSs and Si substrates may allow combining the advantages provided by the features of III–V materials with the well-established and widely used Si technology [38,39,40,41]. The growth technique of artificial GaP/Si substrates suitable for the III–V low-dimensional HSs formation was reported in [42]. The density of threading dislocations in the near-surface layers of the artificial GaP/Si substrate was about 10^8^ cm^−2^.

All HSs were grown by the MBE technique in an improved UHV chamber of the MBE-setup Shtat-type (Ryazan, Russia). It was equipped with crucible sources of fluxes of Al and Ga atoms and Sb_4_ molecules with aperture dampers, as well as two-zone valve sources of P_2_ molecules [43]. The flux densities of P_2_ and Sb_4_ molecules, as well as of Al and Ga atoms, were determined from the values of the ion current of an ionization manometric transducer introduced, during the measurements, into direct fluxes to the substrate position [44]. This method makes it possible to determine the fluxes of atoms of groups III and V with an accuracy of ±2% and ±6%, respectively. The substrate temperature (*T*_s_) was controlled by the thermocouple of the substrate heater, which was calibrated for each sample by the RHEED method in reference to the transition temperatures of surface superstructures on GaP(100) in the absence of a phosphorus flux. This technique was previously developed for the GaAs layer epitaxy [45] and was used here for the GaP growth after small corrections. The temperature determination accuracy was ±5 °C.

**Figure 1:**
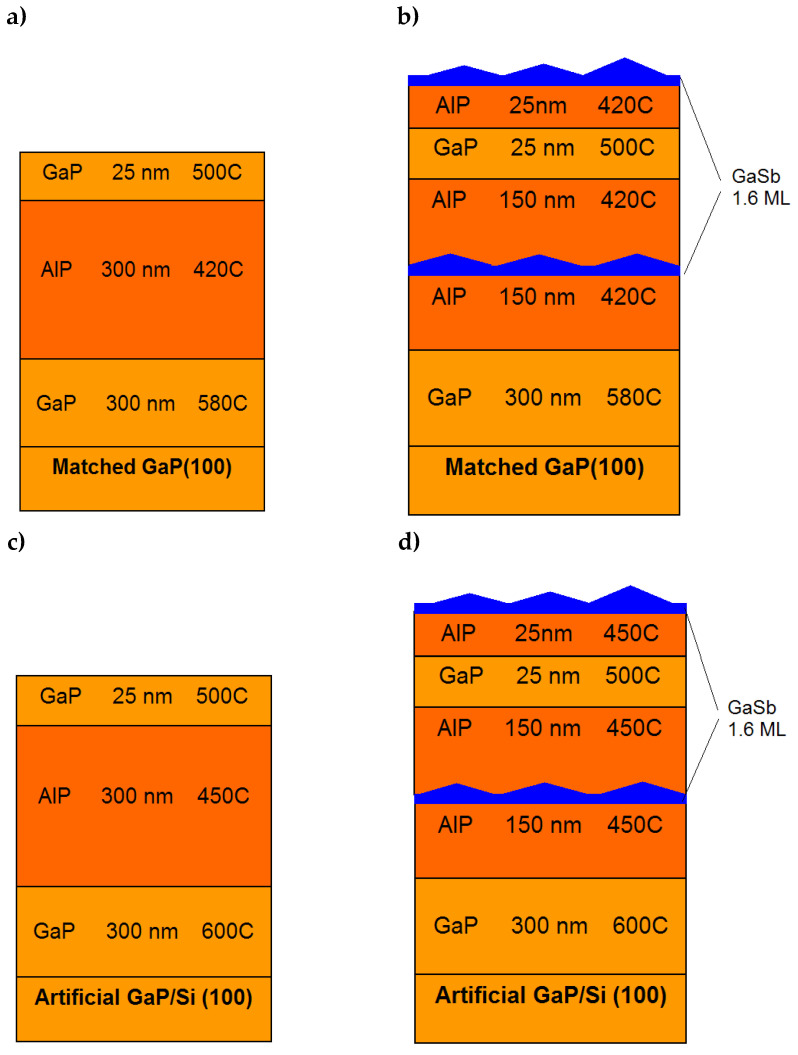
Scheme of epitaxial layers in HSs of various types: (**a**) test HS (**A**) without SAQDs grown on a GaP substrate, (**b**) HSs with SAQDs (type **B**) grown on a GaP substrate, (**c**) test HS without SAQDs (**C**) grown on an artificial GaP/Si substrate, and (**d**) HSs with SAQDs (type **D**) grown on an artificial GaP/Si substrate.

A series of HSs without GaSb/AlP SAQDs (HSs **A** and **C**) and with GaSb/AlP SAQDs (HSs of type **B** and type **D**) was grown on various substrates. The HSs are schematically present in Figure 1. The growth rate of GaP and AlP layers was one monolayer per second (ML/s). The GaP buffer layers with a thickness of 300 nm were grown at *T*_s_ = 580 °C in the HSs grown on a matched GaP substrate. In HSs grown on an artificial GaP/Si substrate, the 300 nm thick GaP buffer layers were grown at *T*_s_ = 600 °C. The buffer layer formation temperature was adjusted to optimize the quality of the growth surface morphology, which was controlled in situ by RHEED. After the buffer layer growth, the *T*_s_ level was decreased to 420 °C for the HSs grown on a matched GaP substrate, and to 450 °C for the HSs grown on an artificial GaP/Si substrate. Then, the AlP layers were grown. The *T*_s_ value for the AlP growth was tuned in accordance with the results of studies on the epitaxial growth of AlP layers [46,47]. The AlP layer thickness was 300 nm in all HSs. The SAQDs were located in the center of the AlP layer, as shown in Figure 1. The SAQDs’ formation was performed at different *T*_s_ values in the range of 360–480 °C. The material deposition rate during SAQD formation was 0.23 ML/s. To induce the SAQDs’ formation, 1.6 MLs of GaSb was deposited. The SAQDs formation was in situ controlled by RHEED. After the deposition of the required amount of material, the growth was interrupted, and the growth surface was kept for 30 s without fluxes of atoms of both groups III and V. The AlP layers were covered by a 25 nm GaP layer grown at the *T*_s_ value gradually increasing from 420 (450 °C) to 500 °C. In the HSs, where SAQDs were formed at 420 °C and 450 °C for HS of type **B** and type **D**, respectively, and 25 nm thick AlP layers were additionally grown, and unburied SAQDs were formed under conditions similar to the conditions used for the growth of buried SAQDs. This was implemented in order to study the SAQD array morphology using the AFM technique.

The SAQDs’ formation process was in situ controlled by the RHEED technique using homemade equipment (ISP SB RAS, Novosibirsk, Russia). The unburied SAQDs array structure was ex situ studied by AFM using a Solver P47 microscope (NT-MDT, Moscow, Russia) operating in the semi-contact mode. The steady-state PL was excited by the radiation from a GaN laser diode (ISP SB RAS, Novosibirsk, Russia) with a photon energy of 3.06 eV (405 nm) and a power density (*P*_ex_) varied in the range 0.37–25 W/cm^2^. The PL radiation was analyzed using an SDL-1 double grating monochromator (LOMO, Saint Petersburg, Russia) and recorded using a nitrogen-cooled Ge photodiode (Edinburgh Instruments, Edinburgh, Great Britain) in the synchronous detection mode. The samples were placed in a helium-filled cryostat Utreks-R (Kharkov, Ukraine), which maintains the temperature in the range of 5–300 K.

## 3. Experimental Results

### 3.1. RHEED

**Figure 2:**
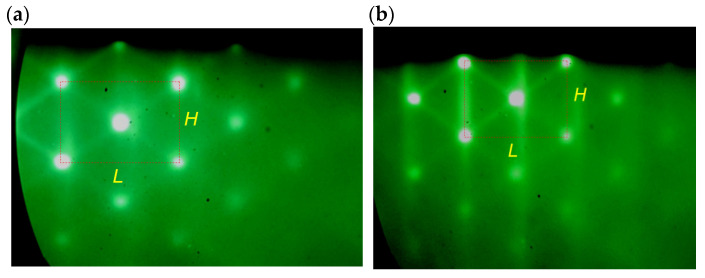
RHEED patterns obtained after the SAQDs formation in the HS of (**a**) type **B** grown at *T*_s_ = 420 °C and in the HS of (**b**) type **D** grown at *T*_s_ = 450 °C. Thin red dotted lines mark the sizes *L* and *H*.

The RHEED pattern of two-dimensional growth was observed during the GaSb deposition until the nominal amount of deposited material reached 1.6 ML. The 2D RHEED pattern was dramatically changed into a typical 3D diffraction pattern when the nominal amount of the deposited material reached 1.6 ML. This transformation indicates the formation of nanosized 3D islands [48,49]. The 3D RHEED patterns corresponded to the SAQDs formation in the HS of type B grown at *T*_s_ = 420 °C and the HS of type D grown at *T*_s_ = 450 °C are shown in Figure 2a,b, respectively. The character of the RHEED patterns was the same for all HSs of type B and type D independently of *T*_s_ variations in the range of 360–480 °C during the SAQDs formation.

An analysis of these RHEED patterns allows us to obtain the in situ information on the strain in the formed SAQDs. It is known that the horizontal distance between diffraction reflections under the conditions of 3D Bragg diffraction is *L* ~ 1/*d*_011_, while the vertical distance is *H* ~ 1/*d*_100_, where *d*_011_ and *d*_100_ are the corresponding interplanar distances in the island material [49]. The distances are governed by the lattice constants of the island material in the corresponding directions: *d*_011_ ~ *a*_||_/2^0.5^ and *d*_100_ ~ *a*_⊥_. Here, *a*_||_ is the lattice constant in the HS plane, and *a*_⊥_ is the lattice constant in the growth direction. In the strained islands, lattice constant values in different directions are not equal to each other, since the compression of the island lattice in the HS plane leads to its expansion in the growth direction [50]. The analysis of the RHEED patterns showed that the *L*/*H* ratio for the HS of type **B** is 1.434, and for the HS of type **D,** it is 1.466, which differs from 2^0.5^ by less than 5% (1.414 for the case of complete strain relaxation). This allows stating that the strain in the obtained SAQDs is almost completely relaxed.

The absence of visible dynamics in the 3D RHEED pattern during the SAQDs’ formation shows that the strain relaxation occurred almost immediately after the SAQDs’ formation and the stage of strained islands is not stable. The variation in *T*_S_ in the range of 360–480 °C during the SAQD formation did not lead to noticeable changes in the SAQD formation process. Regardless of *T*_S_, almost completely relaxed SAQDs are formed. The HS of type **B** with SAQDs grown at 420 °C and the HS of type **D** with SAQDs grown at 450 °C will be discussed further and marked like HS **B** and **D**, respectively.

**Figure 3:**
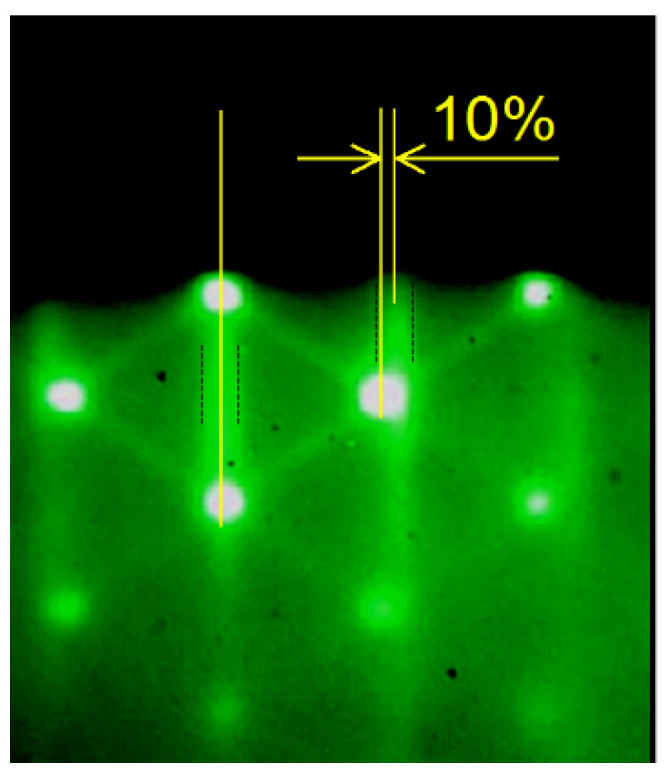
RHEED pattern obtained after the SAQDs formation in HS **D**. The dotted black lines mark the 2D reflections associated with the diffraction on the AlP surface superstructure.

A detailed analysis of the RHEED pattern for HS **D** was performed. Coupled with SAQD 3D Bragg reflections, we observed 2D reflections associated with the electron diffraction on the AlP surface superstructure. The boundaries of these reflections are marked in Figure 3 by vertical black dotted lines. As can be seen in the pattern, the horizontal distances between the zero-order reflection and first-order 3D Bragg reflections and between the zero-order reflection and first-order 2D surface reflections differ by 10 ± 1%. The distance between 2D surface reflections is inversely proportional to the AlP lattice constant, and, therefore, the lattice constant of the SAQD material is 10 ± 1% larger than the AlP lattice constant. Taking into account the mismatch between the lattice constants of GaSb and AlP (10.5% [35]), this also indicates an almost complete strain relaxation into the SAQDs. In addition, these data made it possible to estimate the composition of SAQD material. In general, due to different mixing processes, SAQDs can be formed from the Ga_x_Al_1−x_Sb_y_P_1−y_ quaternary alloy. However, the RHEED results show that the fraction of P atoms in the SAQDs composition is insignificant, since, at a significant element mixing over group V, a proportional change in the alloy lattice constant would occur [35]. Unfortunately, this logic cannot be applied to the mixing of group III materials, since the lattice constants in AlSb/GaSb and AlP/GaP pairs are practically the same [35]. Therefore, we can state that SAQDs consist of an almost unstrained ternary alloy Ga_x_Al_1−x_Sb.

Thus, as a result of RHEED in situ studies of the GaSb/AlP SAQD formation, the growth parameters were determined for the SAQD array formation, and also the data were obtained concerning the strain and chemical composition of the SAQDs.

### 3.2. Atomic Force Microscopy

**Figure 4:**
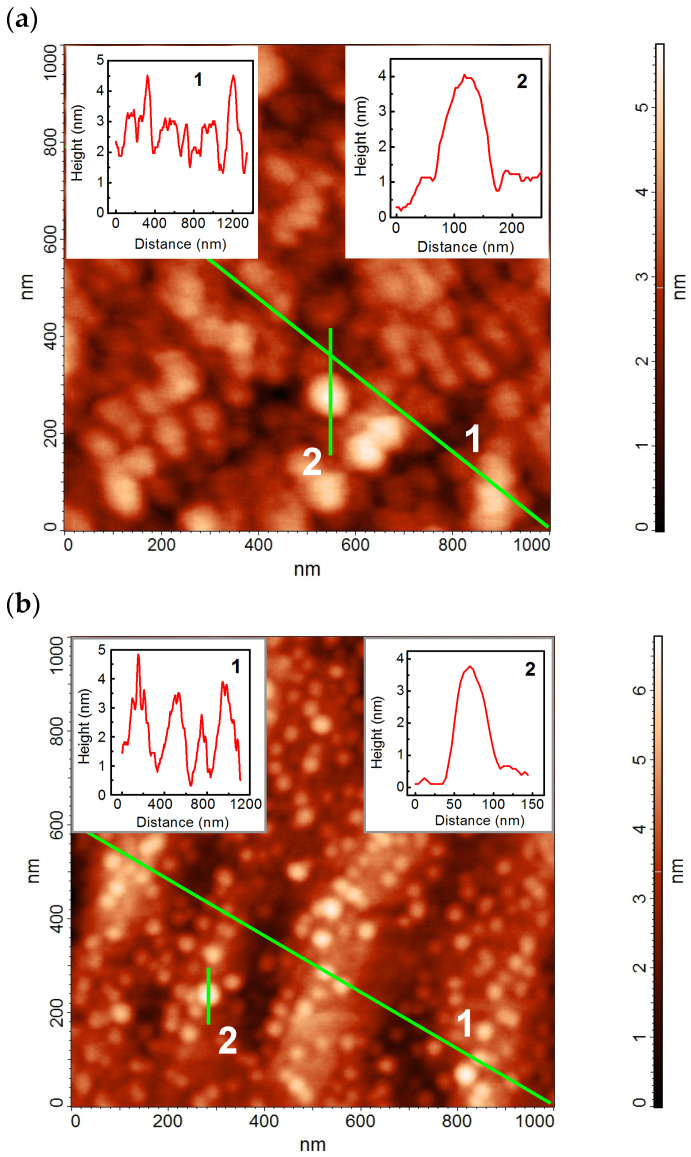
AFM images of the surface of HSs (**a**) **B** and (**b**) **D**. The surface height profiles along different sections are shown in the insets.

The surface of HSs with unburied SAQDs was studied by the AFM technique. AFM scanning was carried out immediately after removing the HSs from the growth chamber in order to minimize the effects of possible AlP interaction with oxygen and water in the atmosphere. The AFM images of the HSs **B** and **D** surface, as well as surface height profiles for various sections, are presented in Figure 4. As can be seen, an SAQD array is actually formed in both HSs. The SAQD array parameters (SAQD density (*N*_QD_), SAQD diameter (*D*_QD_), and SAQD height (*h*_QD_)) are presented in Table 1.

In addition, as it is shown in Figure 4, the AlP surface is characterized by a smooth relief with “waves”. The lateral extension of these “waves” is *D*_AlP_ and the vertical magnitude is *h*_AlP_. The values of these parameters are also presented in Table 1.

In our opinion, an increase in the *h*_AlP_ value for the smooth AlP relief in the GaP/Si-based HS, in comparison with the GaP-based HS, can be caused by (1) a larger RMS of the initial GaP growth surface in the case of artificial GaP/Si substrate; and (2) the influence of threading dislocations, which are present in the layers grown on an artificial GaP/Si substrate. An increase in the SAQD array *N*_QD_ is observed in parallel with an increase in the vertical magnitude of the smooth AlP relief *h*_AlP_, while the lateral extension of the AlP relief is approximately the same. We assume that this effect can be attributed to an increase in the atomic step density on the AlP surface and, thus, to an increase in the number of places where the SAQD formation is preferable. Note, that an approximately twofold increase in *h*_AlP_ (and steps density also) results in a proportional increase in *N*_QD,_ and this is in favor of our suggestion. The same assumption can be used to explain the decrease in the *D*_QD_ [51]. Moreover, an effect of the threading dislocation, which is present in GaP/Si-based HSs, on the SAQD formation process, cannot be excluded. It is possible that the dislocation outcrops play the role of a source of mobile adatoms because of the crystal structure distortion in the dislocation core. It can have an effect on lateral adatom diffusion during the SAQDs’ formation. Unfortunately, our experimental data are not enough for a correct estimation of the contribution of each discussed mechanism. Therefore, this question is out of the scope of this study.

It is necessary to note that the SAQD arrays are characterized by a sufficiently high density (several units of 10^10^ cm^−2^), and a high charge plane density may be expected when the SAQD states are filled with holes. This is useful from the point of view of the future application of these HSs as floating gates in memory cells since it will increase the efficiency of the SAQD layer effect on the underlying FET channel conductivity.

Thus, the ex situ AFM studies of an array of unburied GaSb/AlP SAQDs grown on various substrates made it possible to obtain information on the AlP surface morphology used for the SAQD array formation, as well as on the geometric parameters of the SAQD arrays.

### 3.3. Photoluminescence

**Figure 5:**
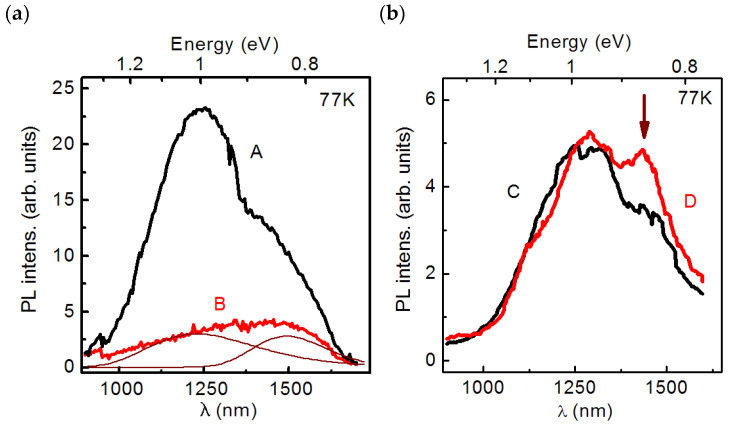
Steady-state PL spectra of HSs (**a**) **A** and **B**, and (**b**) **C**, and **D**, measured under the nonresonant excitation in an AlP matrix at the temperature of 77 K and *P*_ex_ = 25 W/cm^2^. Thin brown lines in figure (**a**) show the PL spectrum deconvolution to Gaussians.

The steady-state PL spectra of all considered HSs were measured under the nonresonant excitation of nonequilibrium charge carriers in an AlP matrix at the liquid nitrogen temperature (77 K) and *P*_ex_ = 25 W/cm^2^. The measured results are shown in Figure 5.

**Figure 6:**
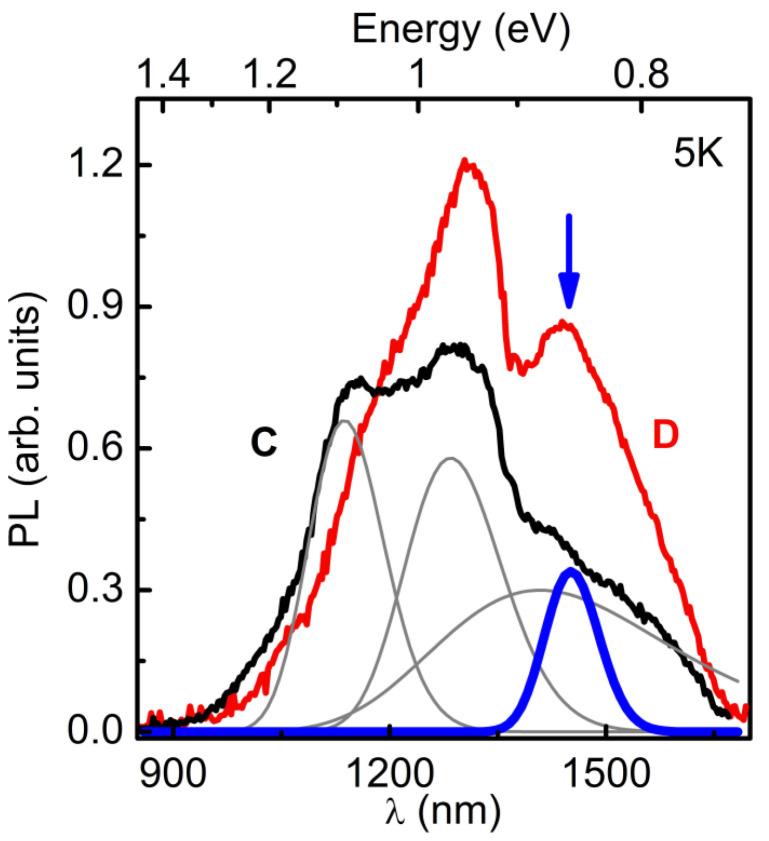
Steady-state low-temperature (5 K) PL spectra measured under the nonresonant excitation in an AlP matrix at *P*_ex_ = 25 W/cm^2^ for the GaSb/AlP SAQD HS **D** (red line) and HS **C** without SAQDs (black line). Thin gray lines show a set of Gaussians with the maxima at the energies of 0.88, 0.96, and 1.09 eV used to fit the spectrum of HS **C**. In the PL spectrum of HS **D**, an additional PL band appears with a maximum at 0.86 eV, as shown by a thick blue line and by a blue arrow.

**Figure 7:**
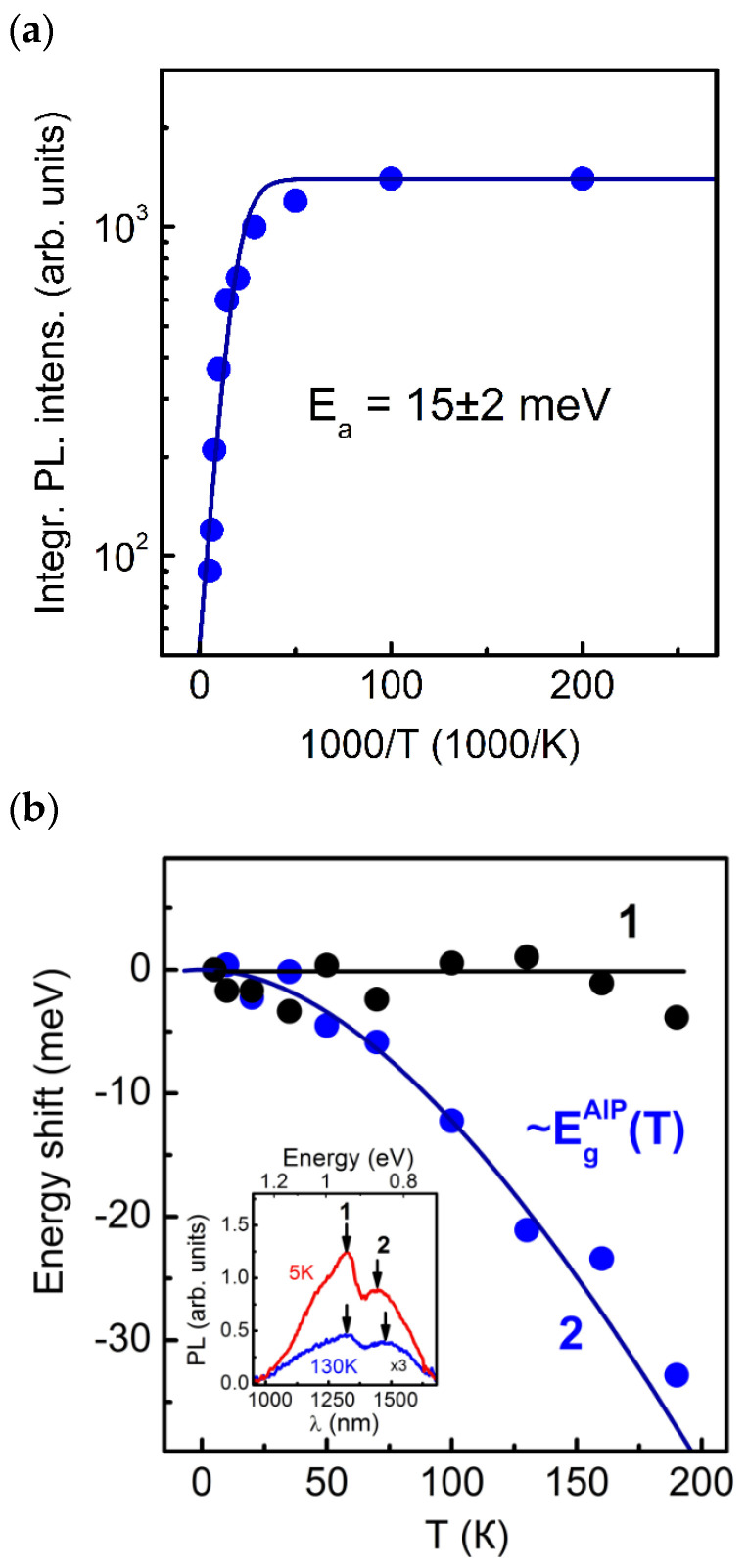
(**a**) Dependence of the integral SAQDs PL intensity on the temperature. The measurements were carried out at *P*_ex_ = 25 W/cm^2^. The temperature quenching of PL is described by the Arrhenius function with the activation energy of 15 ± 2 meV. (**b**) Temperature dependences of the energy shift in the PL bands with the maxima at 0.95 eV (1) and 0.86 eV (2). The solid lines show the functional approximation experimental data. The PL spectra of HS **D** measured at 5 and 130 K are shown in the inset. The arrows and numbers 1 and 2 indicate the positions of the maxima of the PL bands associated with the defects in AlP and SAQDs, respectively.

**Figure 8:**
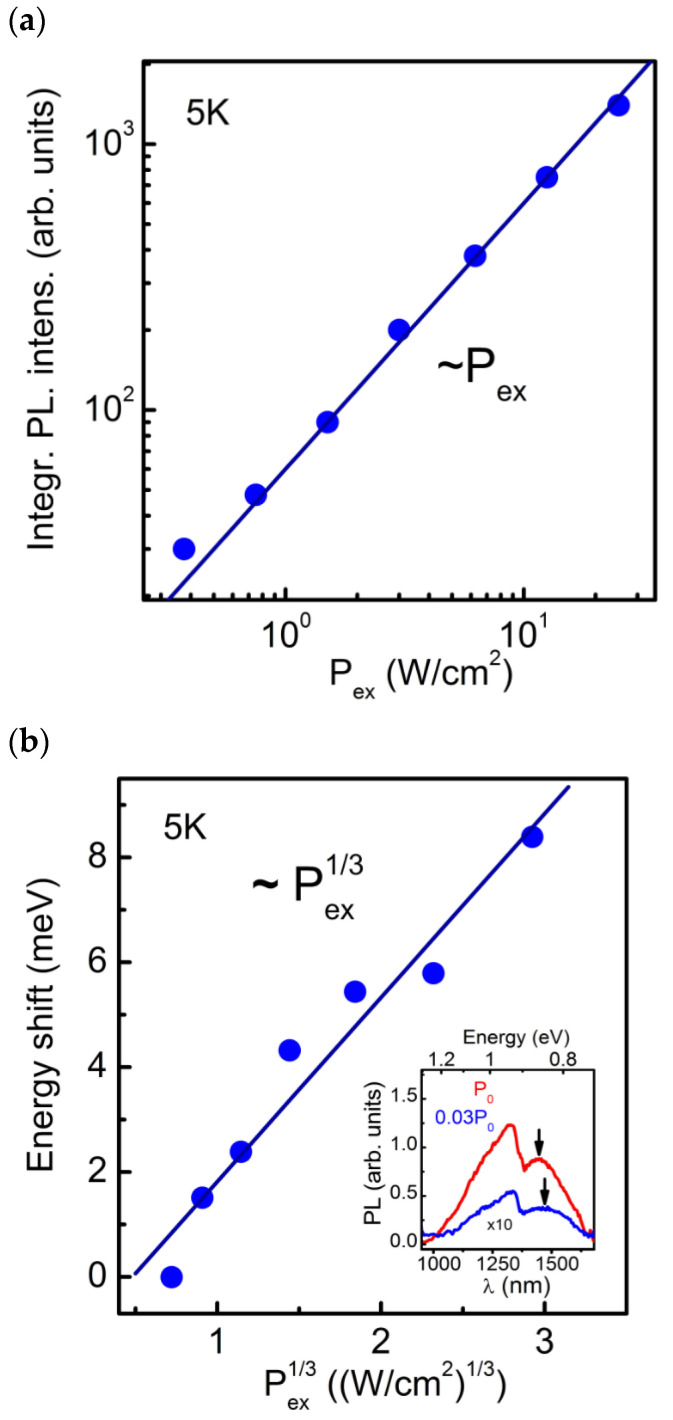
(**a**) Dependence of the integral SAQD PL intensity on *P*_ex_. The measurements were carried out at a temperature of 5 K. The solid line shows the approximation of the experimental data by the ~*P*_ex_ law. (**b**) The energy shift in the SAQD PL band as a function of *P*_ex_. The solid line shows the approximation by the law ~*P*_ex_^1/3^. In the inset are the PL spectra of HS **D** measured at *P*_ex_ = 25 W/cm^2^ (red curve) and *P*_ex_ = 0.75 W/cm^2^ (blue curve). The arrows mark the positions of the SAQDs PL band maxima.

As it is presented in Figure 5a, the spectrum of HS A contains two PL bands with the maxima at energies 1.01 and 0.83 eV. We attribute these bands to the charge carrier recombination through deep centers in the AlP and/or GaP layers. The spectrum of HS B also contains both PL bands with the maxima at energies 1.01 and 0.83 eV, as can be seen from the deconvolution of the PL spectrum of HS B into the sum of two Gaussian components (Figure 5a). Unfortunately, no additional PL bands that could be associated with the charge carrier recombination in SAQDs were detected. It is also necessary to note that the integrated PL intensity of HS B is noticeably (four times) lower than the integrated PL intensity of HS A. At the same time, a comparison of the PL spectra of HSs C and D presented in Figure 5b made it possible to reveal the PL band which is, probably, associated with the charge carrier recombination in SAQDs. This band with a maximum at 0.85 eV is marked with a vertical arrow in Figure 5b. Detailed studies of the luminescence properties of HSs B and D were carried out. The low-temperature (5 K) steady-state PL spectra of the HSs were measured, and the dependences of the PL spectra on temperature and *P*_ex_ were analyzed. The obtained results are presented in Figure 6, Figure 7 and Figure 8.

As it is presented in Figure 6, the spectrum of HS C without SAQD consists of bands whose shape is well described by Gaussian functions with the maxima at the energies of 0.88, 0.96, and 1.09 eV, with the full width at half maximum (FWHM) values of 230, 120, and 110 meV, respectively. We attribute these PL bands to the charge carrier recombination through deep levels in the AlP and/or GaP layers. A detailed elucidation of the nature of these deep levels is beyond the scope of this work. The spectrum of SAQD HS D contains PL bands, whose shape is also described by the Gaussian function, and their spectral position, the FWHM and amplitude values are close to the corresponding bands in the spectrum of the HS C without SAQDs. The bands are characterized by the maxima at energies of 0.88, 0.95, and 1.08 eV, with FWHM values of 230, 112, and 120 meV, respectively. The change in the PL band intensity compared to the spectrum of the HS without SAQDs is no more than 30%. We also attribute these PL bands to the recombination through deep levels in the bulk material. Furthermore, the spectrum contains an additional PL band with a maximum at 0.86 eV and an FWHM value of 72 meV, which is marked in Figure 6 with a thick blue line. We attribute this additional band to the charge carrier recombination in the SAQDs. As can be seen, the PL data obtained at 5 K are in good agreement with the PL data obtained at 77 K (Figure 5).

The PL spectra of SAQD HS D were measured at different temperatures in the range of 5–190 K. The measurements were carried out at *P*_ex_ = 25 W/cm^2^. The results are shown in Figure 7. As the temperature increases, the SAQD PL quenches. The temperature dependence of the integrated SAQD PL intensity is shown in Figure 7a. The experimental dependence is well described by the Arrhenius function $\frac{A}{1+B\cdot e^{-\frac{E_{a}}{kT}}}$ with the PL quenching activation energy *E*_a_ = 15 ± 2 meV. As is shown further by the energy spectrum calculation, the value of *E*_a_ is due to the fact that the SAQD PL quenching occurs due to the nonequilibrium electron ejection from the SAQD into the AlP matrix. A shift in the spectral position of the PL bands is also observed. In Figure 7b are the dependences of the spectral shift magnitude of the PL bands with the maxima at 0.95 eV, marked with black dots and number 1, and with a maximum at energy of 0.86 eV, marked with blue dots and the number 2. As can be seen from the curves, the SAQD PL band at 0.86 eV shows a shift that is well described by the temperature dependence of the AlP bandgap. However, the PL band at 0.95 eV is almost not shifted with temperature. The bands with the maxima at 0.88 eV and 1.08 eV behave in a similar way. The observed character of the temperature dependences of the integrated PL intensity and the spectral shift in PL bands, additionally, indicates that the PL band with the maximum at 0.86 eV is associated with the charge carrier recombination in SAQDs, while the remaining spectral bands are caused by the recombination on deep levels in AlP and/or GaP.

The PL spectra of the SAQD HS were measured at the temperature of 5 K and *P*_ex_ varying in the range of 0.375–25 W/cm^2^. The measured results are shown in Figure 8. As can be seen in Figure 8a, the integral SAQD PL intensity is nearly proportional to *P*_ex_. According to the results of [52], this indicates that the internal quantum yield of radiative recombination of nonequilibrium charge carriers in the SAQDs is close to 100%, and almost all electron-hole pairs captured into the SAQDs recombine radiatively. In addition, a spectral shift in the SAQD PL band is observed with the *P*_ex_ increasing. The energy shift dependence on *P*_ex_ is shown in Figure 8b. As can be seen from the curves, the SAQDs PL band energy shift is proportional to *P*_ex_^1/3^. In accordance with the results reported in [53,54,55], this indicates that the SAQDs have a band alignment of type II, which implies the spatial separation of electrons and holes localized in the SAQDs. The PL band shift is caused by the energy band distortion, and, as a consequence, the shift in the quantum confinement levels with a change in the number of charge carriers localized in the SAQDs.

Thus, the PL experiments made it possible to unambiguously identify the PL band associated with the recombination of nonequilibrium charge carriers in the SAQDs grown on artificial GaP/Si substrates. We list here the main experimental factors, which allow us to state that the founded PL band is associated with the SAQDs:

1. This PL band exists in the spectrum of HS **D** with SAQDs and is absent in the spectrum of HS **C** without SAQDs.
2. This PL band shifts towards lower energies with increasing temperature (Figure 7b). This behavior is opposite to deep-level PL bands (0.8–1 eV), which have a stable energy position. Moreover, the SAQD PL band shift is in excellent relation to the temperature dependence of E_g_ of AlP. It indicates that PL energy is governed by the energy level position in the SAQD.
3. SAQD PL band shits ~ *P*_ex_^1/3^ when excitation density *P*_ex_ is changed. This behavior is peculiar to low-dimensional systems with band alignment of type II [53,54,55] and not peculiar to deep-level PL.

### 3.4. Summarizing Experimental Results

At the end of the section, we would like to list the most important experimental results, in our opinion. The GaSb/AlP HSs were studied by RHEED, AFM, and steady-state PL spectroscopy. As a result, it was shown:

1. The deposition of 1.6 ML GaSb on the AlP surface at *T*_S_ = 360–480 °C, where AlP was grown on matched GaP, as well as on artificial GaP/Si substrates, leads to the SAQD array formation.
2. An almost complete strain relaxation is observed in the SAQDs regardless of the substrate type employed for the SAQD growth.
3. SAQDs grown on an artificial GaP/Si substrate consist of almost unstrained Ga_x_Al_1−x_Sb.
4. The geometric parameters of the SAQD arrays were determined, as well as their correlations with the morphology of the AlP surface on which the SAQDs were formed (Table 1): an increase in the AlP surface roughness leads to an increase in the SAQDs density and a decrease in their sizes.
5. No PL bands that could be associated with the recombination of nonequilibrium charge carriers in SAQDs grown on matched GaP substrates were found.
6. The SAQD formation in HSs grown on matched GaP substrates leads to a significant (fourfold) decrease in the integrated intensity of PL bands associated with the nonequilibrium charge carrier recombination through deep levels in AlP and/or GaP.
7. A low-temperature PL band with a maximum at about 0.86 eV was found, and the band is associated with the nonequilibrium charge carrier recombination in GaSb/AlP SAQDs grown on artificial GaP/Si substrates.
8. The thermal quenching of the SAQD PL occurs at *E*_a_ = 15 ± 2 meV.
9. SAQDs grown on an artificial GaP/Si substrate are characterized by a high internal luminescence quantum yield close to 100%.
10. A spectral shift in the PL band of SAQDs grown on an artificial GaP/Si substrate was observed. This shift is ~*P*_ex_^1/3^, and it indicates that SAQDs have a band alignment of type II.

## 4. Discussion

In this section, we discuss the structural properties of the considered GaSb/AlP SAQDs, as well as the SAQD energy spectrum.

### 4.1. Structural Properties of SAQDs

**Figure 9:**
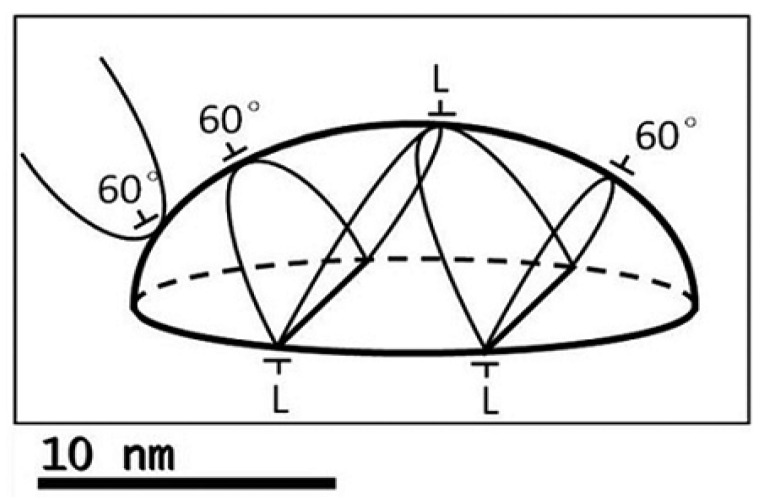
Location of dislocation loops around the SAQD; 60° dislocations tend to combine into Lomer dislocations (L). A dislocation loop (left) fails to wrap around the dot and threads toward the surface [62].

The main feature of the SAQD structure is an almost complete absence of strain, as indicated by the RHEED data. Strains caused by a mismatch between the lattice constants of the deposited material and the matrix material are one of the main reasons for the SAQDs’ formation. The reorganization of the surface structure and the formation of 3D islands (SAQDs) leads to a decrease in the total energy of the system due to a decrease in the elastic energy in SAQDs, despite an increase in the surface energy [51,56,57]. However, an increase in the SAQDs’ size leads to an increase in the elastic energy stored in them. When the elastic energy reaches a threshold value, the plastic relaxation of strains in SAQDs occurs by the introduction of dislocations [58,59,60,61,62,63]. It is known that the coalescence of two complementary 60° dislocations (U-half-loops) leads to the formation of a complex containing a Lomer 90° dislocation lying in the (001) plane and threading arms lying in planes of the (111) type [64,65,66,67]. Sliding of threading arms in planes of type (111) leads to their going beyond the SAQD, and, thus, only a 90° dislocation remains inside and in the vicinity of the SAQD. This dislocation does not cross the SAQD volume. The schematic image of the dislocation complex in the SAQD when threading arms exist in the SAQD volume is presented in Figure 9. As shown in [68], the core of the Lomer dislocation segment does not contain uncompensated atomic bonds, unlike threading arms. Therefore, the presence of 90° dislocations does not lead to the formation of deep centers and, consequently, to an increase in the probability of nonradiative recombination in SAQDs. We have already observed a similar effect in GaSb/GaP [69] and GaAs/GaP [70,71] heterosystems. At the same time, if no nucleation and effective coalescence of complementary 60° dislocations occurred during the formation of the SAQD and its plastic relaxation, then 60° dislocations remain in the SAQD. This leads to a sharp quenching of PL due to an increase in the rate of nonradiative recombination. It is necessary to note that in both cases the threading arms are growing from the SAQD into the volume of the AlP matrix and affect the concentration of photoexcited nonequilibrium charge carriers by increasing the rate of nonradiative recombination. However, the absence of nonradiative centers directly in SAQDs, where Lomer dislocations are formed, makes it possible to observe the PL of such SAQDs. Our PL data show that the PL of GaSb/AlP SAQDs grown on an artificial GaP/Si substrate is characterized by a high internal PL quantum yield (about 100%), and it indicates that the introduction of dislocations had no noticeable effect on the efficiency of radiative recombination of nonequilibrium charge carriers.

Thus, it can be suggested that the strain relaxation occurs due to the introduction of Lomer dislocations located at the SAQD/matrix heterointerface in the GaSb/AlP HSs grown on artificial GaP/Si substrates. At the same time, the absence of an SAQD PL band for GaP-based HSs, as well as a decrease in the PL associated with the recombination at deep levels in AlP and/or GaP as a result of the SAQDs formation, suggests that the strain relaxation in GaP-based SAQDs is provided by the introduction of 60°-dislocations penetrating the SAQD volume. Today, available experimental data are not enough to unambiguously reveal the reason for such a drastic change in the strain relaxation mode; however, we assume that this may be caused by the presence of threading dislocations in the volume of GaP and AlP layers in HSs grown on artificial GaP/Si substrates. Indeed, the presence of threading arm dislocations in GaP and AlP layers grown on a GaP/Si substrate can facilitate efficient coalescence of 60° dislocations formed during SAQD formation. This assumption is supported by the results obtained in [67], where it was shown that the presence of an initial density of threading dislocations in a relaxing layer has a significant effect on the mechanism of introducing dislocations and leads to an increase in the probability of the formation of the complementary pairs of 60° dislocations.

### 4.2. Energy Spectrum

**Figure 10:**
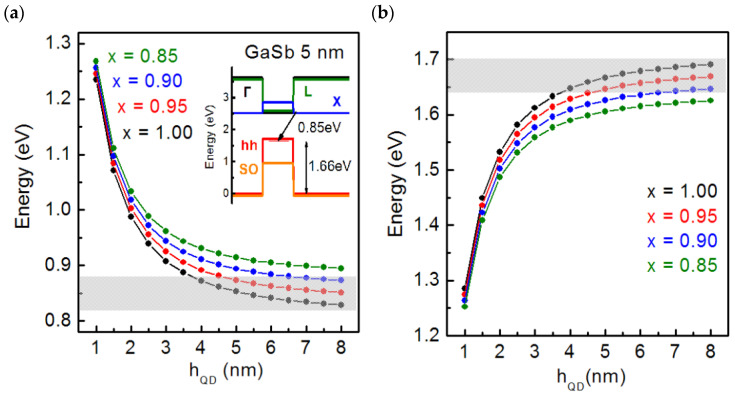
(**a**) Calculated optical transition energy dependences on the Ga_x_Al_1−x_Sb/AlP SAQD height for various alloy composition values. In the inset is the band diagram for GaSb/AlP SAQDs with a height of 5 nm. The shaded area corresponds to the SAQD optical transition energy values obtained from experimental PL data. (**b**) Calculated *E*_loc_ dependences on the Ga_x_Al_1−x_Sb/AlP SAQD height for different alloy compositions. The shaded area corresponds to the *E*_loc_ values obtained from a comparison of the PL data and the calculation results.

In order to determine the energy spectrum of the SAQDs, in particular the *E*_loc_ value, the SAQD energy spectrum was calculated and the results were compared with the experimental PL data. Following the results of [29,33], we use a truncated tetrahedral pyramid for modeling a SAQD shape. According to the AFM data, the pyramid base length-to-height ratio is equal to 15:1. Note that obtaining precise information about the SAQD shape from the AFM data is complicated by the convolution effect during scanning the surface features of sizes comparable with the probe tip radius. Thus, the SAQD is modeled as a truncated pyramid consisting of a Ga_x_Al_1−x_Sb alloy of uniform composition. Since available experimental data did not allow the obtaining of information about the Ga (Al) content in the SAQDs, different alloy compositions were considered. The Ga_x_Al_1−x_Sb alloy parameters were estimated from the known parameters of GaSb and AlSb within the quadratic approximation [35]:

(1) $$P_{\mathrm{GaAlSb}}=x\cdot P_{\mathrm{GaSb}}+\left(1-x\right)\cdot P_{\mathrm{AlSb}}+x\left(1-x\right)\cdot C_{\mathrm{GaAlSb}},$$

where *P*_GaSb_ and *P*_AlSb_ are the values of the corresponding parameters for GaSb and AlSb, and *C*_GaAlSb_ is the bowing parameter. Calculations of the energy level positions for electrons and holes were implemented in the framework of the single band approximation, and the exciton effect was not taken into account. The variations in the SAQD sizes were also accounted for in the calculations. A detailed discussion of the approaches used in the calculations can be found in our previous work [36] related to the calculations of the InGaSb/AlP SAQDs energy spectrum. The values of the AlP, GaSb, and AlSb material parameters, such as the band gap at the Г, X, and L points of the Brüllien zone; spin-orbit splitting value in the valence band; valence band offset (VBO); and charge carrier effective masses, as well as the corresponding bowing parameters, were reported in [35,72]. The material parameters used in the calculations are presented in Table 2. The calculations were performed using the Nextnano++ program package [73]. This program package is commonly used for III–V SAQD energy spectrum calculations [28,31]. The results are shown in Figure 10.

The calculations show that, independently from the SAQD composition and sizes, the ground electronic state lies in the *X* valley of the AlP conduction band, and the ground hole state lies in the heavy hole subband of Ga_x_Al_1−x_Sb. This is in excellent agreement with our experimental PL data, which indicate a type-II band alignment for the SAQDs. In addition, the observed *E*_a_ of the SAQD PL temperature quenching is 15 ± 2 meV, which is in good agreement with the weak localization of electrons in the vicinity of the SAQD of type II.

The calculated dependences of the SAQD optical transition energy are shown in Figure 10a. An increase in the SAQD sizes leads to a decrease in the optical transition energy, which is explained by the quantum confinement effect. An increase in the content of Al atoms in the SAQD composition leads to an increase in the optical transition energy, which is caused by an increase in the Ga_x_Al_1−x_Sb alloy bandgap. The comparison of the calculated optical transition energy values with the experimental PL data (shaded area in Figure 10a) is performed taking into account the PL bandwidth. It reveals the range of admissible values of SAQD sizes and composition. As can be seen from the curves, the SAQD cannot contain more than 10% Al in the composition, and the SAQD height cannot be less than 4 nm. At the same time, our AFM data show that the vertical SAQD sizes do not exceed 4 nm. Possible reasons for this discrepancy are now discussed. The underestimation of the SAQD height in the AFM measurements can be caused by the following reasons: (1) the effect of convolution in the AFM measurements of surface morphology when the sizes of features are comparable with the probe tip radius [74]; (2) SAQDs’ degradation during the HS cooling with unburied SAQDs in the residual atmosphere of the growth chamber; the time of which noticeably exceeds the growth pause time for buried SAQDs; and (3) the formation of an oxide layer on the surface during the HS exposure to the atmosphere.

As seen in Figure 10b, the performed calculations show that the *E*_loc_ value in Ga_x_Al_1−x_Sb/AlP is 1.65–1.70 eV, depending on the composition and size of SAQDs. According to [37], the hole storage time can be estimated by:

(2) ts=eEakTγT2σinf

where *T* is the temperature, *σ*_inf_ is the capture cross-section at a high temperature and γ is the coefficient independent of temperature. Localization energies of 1.65–1.70 eV are high enough for the hole storage times of >>10 years, at typical *σ*_inf_ values of 10^−12^–10^−9^ cm^2^ for GaSb/GaP [32], InGaAs/GaP [30], and InGaSb/GaP [33] SAQDs, according to the available experimental results. It makes the Ga_x_Al_1−x_Sb/AlP SAQDs a promising object for the fabrication of universal memory cells.

## 5. Conclusions

The formation processes, structural properties, and energy spectrum of novel GaSb/AlP HSs with SAQDs grown by MBE on matched GaP and artificial GaP/Si substrates were studied. Artificial substrates were characterized by a threading dislocation density of about 10^8^ cm^−2^. The growth conditions were found to form the SAQD arrays on the AlP layers grown on different substrates. It was found that the strains in the SAQDs relax almost completely independently from the substrate type. The strain relaxation in GaP/Si-based SAQDs does not lead to a decrease in the SAQD luminescence efficiency, while the dislocations present in GaP-based SAQDs lead to a strong decrease in the SAQD luminescence efficiency. Probably, this is caused by the introduction of Lomer 90°-dislocations without uncompensated atomic bonds in GaP/Si-based SAQDs, while threading 60°-dislocations are introduced into GaP-based SAQDs. It was shown that SAQDs grown on an artificial GaP/Si substrate consist of an almost unstrained Ga_x_Al_1−x_Sb alloy with a fraction of Al atoms not exceeding 10%. The comparison of experimental PL data and calculation results made it possible to reveal that SAQDs have a band alignment of type II with an indirect bandgap and the ground electronic state belonging to the *X*-valley of the AlP conduction band and the ground hole state lying in Ga_x_Al_1−x_Sb. The estimated *E*_loc_ value was in the range of 1.65–1.70 eV. This allows us to predict the charge storage time in SAQDs at room temperature to be as long as >> 10 years, and that makes these SAQDs a promising object for the fabrication of universal memory cells.

## Figures and Tables

**Table 1:** Parameters of the QD array formed in HSs **B** and **D**, as well as the AlP surface relief parameters in these structures.

Parameter	B, on GaP	D, on GaP/Si
*N*_QD_	1 × 10^10^ cm^−2^	2.5 × 10^10^ cm^−2^
*D*_QD_	50–100 nm	30–60 nm
*h*_QD_	2–3 nm	2–4 nm
*D*_AlP_	200–400 nm	200–400 nm
*h*_AlP_	1–1.5 nm	2.5–3 nm

**Table 2:** Material parameters for AlP, AlSb, GaSb, and the bowing parameter for Ga_x_Al_1−x_Sb at 5 K, which were used for the calculations. *E*_g_^Г,X,L^—bandgaps for Г-, X-, and L-valleys; Δ_0_—spin-orbital splitting energy in the valence band; *VBO*—valence band offsets; *m*_Г_—effective electron mass in the Г-point of the Brillouin zone; *m*_X_^t^ and *m*_X_^l^—transversal and longitudinal effective electron mass in the X-point of the Brillouin zone; *m*_L_^t^ and *m*_L_^l^—transversal and longitudinal effective electron mass in the L-point of the Brillouin zone; *m*_hh_, *m*_lh_, and *m*_SO_—effective masses for heavy, light, and spin-orbital splitting holes, respectively.

Parameter	AlP	AlSb	GaSb	C_GaAlSb_
*E*_g_^Г^, eV	3.63 ^a^	2.386 ^a^	0.812 ^a^	−0.044 + 1.22*x* ^a^
*E*_g_^X^, eV	2.52 ^a^	1.696 ^a^	1.141 ^a^	0 ^a^
*E*_g_^L^, eV	3.57 ^a^	2.329 ^a^	0.875 ^a^	0 ^a^
Δ_0_, eV	0.07 ^a^	0.676 ^a^	0.76 ^a^	0.3 ^a^
*VBO*, eV	−1.74 ^a^	−0.41 ^a^	−0.03 ^a^	0 ^a^
*m*_Г_	0.22 ^a^	0.14 ^a^	0.039 ^a^	0 ^a^
*m*_X_^t^	0.155 ^a^	0.123 ^a^	0.22 ^a^	0 ^a^
*m*_X_^l^	2.68 ^a^	1.357 ^a^	1.51 ^a^	0 ^a^
*m*_L_^t^	0.15 ^b^	0.23 ^a^	0.10 ^a^	0 ^a^
*m*_L_^l^	1.2 ^b^	1.64 ^a^	1.3 ^a^	0 ^a^
*m*_hh_	0.63 ^c^	0.8 ^c^	0.34 ^c^	0 ^a^
*m*_lh_	0.20 ^c^	0.13 ^c^	0.0447 ^c^	0 ^a^
*m*_SO_	0.30 ^a^	0.22 ^a^	0.12 ^a^	0 ^a^

^a^ [35], ^b^ GaP value, ^c^ [72].

## Data Availability

The data are available from the authors on request.
